# The ‘speck’-tacular oversight of the NLRP3-pyroptosis pathway on gastrointestinal inflammatory diseases and tumorigenesis

**DOI:** 10.1186/s12929-023-00983-7

**Published:** 2023-10-27

**Authors:** Valentina Arrè, Rosanna Scialpi, Matteo Centonze, Gianluigi Giannelli, Maria Principia Scavo, Roberto Negro

**Affiliations:** 1 Personalized Medicine Laboratory, National Institute of Gastroenterology “S. de Bellis”, IRCCS Research Hospital, Via Turi 27, 70013 Castellana Grotte, BA Italy; 2 Scientific Direction, National Institute of Gastroenterology “S. de Bellis”, IRCCS Research Hospital, Via Turi 27, 70013 Castellana Grotte, BA Italy

**Keywords:** Inflammasome, NLRP3, Gasdermin D, Pyroptosis, Intestinal Bowel Disease, Gastrointestinal cancer

## Abstract

The NLRP3 inflammasome is an intracellular sensor and an essential component of the innate immune system involved in danger recognition. An important hallmark of inflammasome activation is the formation of a single supramolecular punctum, known as a speck, per cell, which is the site where the pro-inflammatory cytokines IL-1β and IL-18 are converted into their bioactive form. Speck also provides the platform for gasdermin D protein activation, whose N-terminus domain perforates the plasma membrane, allowing the release of mature cytokines alongside with a highly inflammatory form of cell death, namely pyroptosis. Although controlled NLRP3 inflammasome-pyroptosis pathway activation preserves mucosal immunity homeostasis and contributes to host defense, a prolonged trigger is deleterious and could lead, in genetically predisposed subjects, to the onset of inflammatory bowel disease, including Crohn's disease and ulcerative colitis, as well as to gastrointestinal cancer. Experimental evidence shows that the NLRP3 inflammasome has both protective and pathogenic abilities. In this review we highlight the impact of the NLRP3-pyroptosis axis on the pathophysiology of the gastrointestinal tract at molecular level, focusing on newly discovered features bearing pro- and anti-inflammatory and neoplastic activity, and on targeted therapies tested in preclinical and clinical trials.

## Background

**Fig. 1:**
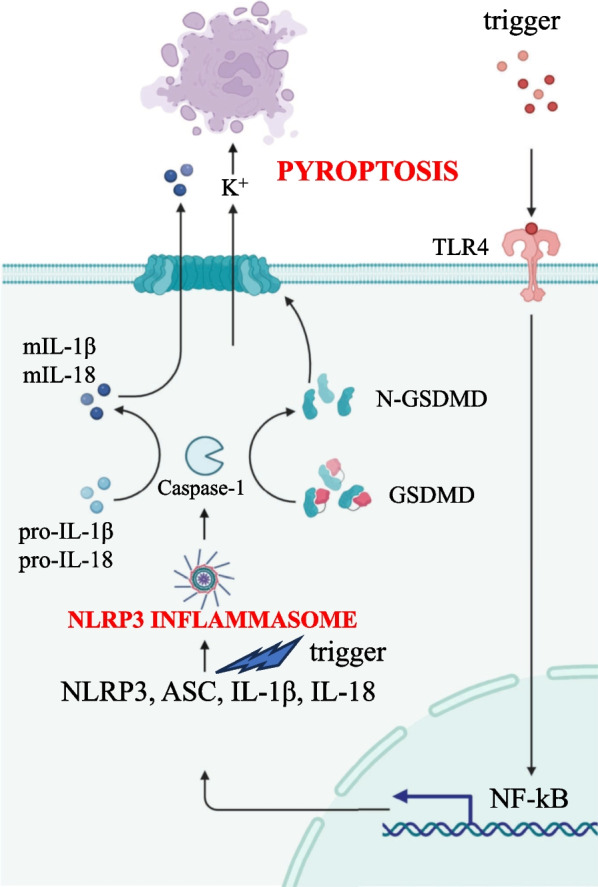
Scheme of the molecular mechanism of the NLRP3 inflammasome-pyroptosis pathway. The canonical NLRP3 inflammasome activation involves two consequential steps, the priming step, where triggers signal to NF-κB via TLR4 promoting the expression of NLRP3, ASC, pro-IL-1β and pro-IL-18, and the activation step, where a second trigger fosters its fully activation determining Caspase-1 cleavage, maturation of IL-1β (mIL-1β), IL-18 (mIL-18) and GSDMD (N-GSDMD). N-GSDMD auto-assemble into plasma membrane eventually leading to pyroptosis and cytokine release

Pattern recognition receptors (PRRs) pose the first eukaryotic line of defense against pathogens, being essential components of the innate immune response against sterile and non-sterile insults [1]. Under stress conditions or infection, PRRs sense and neutralize microbial elements, known as pathogen-associated molecular patterns (PAMPs), as well as several components derived from damaged or dying cells, named damage-associated molecular patterns (DAMPs) [2]. Upon recognition of specific ligands, PRRs transduce the signal to a cytosolic multiprotein complex denominated the inflammasome, which, in turn, triggers the inflammatory response [3]. Well characterized examples of PRRs include the Nucleotide-binding oligomerization domain, Leucine rich Repeat (NLR) and Pyrin (NLRP) or Card (NLRC) domain containing inflammasomes, absent in melanoma-2 (AIM2)-like receptor (ALR)-associated inflammasome, and Pyrin inflammasome expressed in both immune (e.g., monocytes, macrophages, dendritic cells) and non-immune cells (e.g., intestinal epithelial cells, fibroblasts) [4], that can detect a vast range of microbial inputs [5]. The human NLR family includes 22 members, the best characterized of which are NLRP1, NLRP3 and NLRC4, alongside the AIM2 inflammasome [6]. Upon sensing and binding different threats, PRRs signal to an adaptor protein, named apoptosis-associated speck-like protein containing a CARD (ASC) which, in turn, activates pro-Caspase-1, forming the core of the inflammasome. Once activated, Caspase-1 cleaves its three substrates, pro-IL-1β, pro-IL-18 and gasdermin D (GSDMD) into their bioactive form. The N-terminus GSDMD assemble into pores that migrate, together with nerve injury-induced protein 1, named ninjurin-1 (NINJ1), to the plasma membrane allowing the release of mature cytokines IL-1β and IL-18 into the bloodstream, together with a form of inflammatory cell death called pyroptosis (Fig. 1). A common hallmark of inflammasome activation is the formation of a single, cytosolic, supramolecular structure (also known as a punctum or “speck”) per cell, ranging from 1 to 3 μm in diameter and localizing at the centrosome in the perinuclear region [7]. Despite its protective function, inflammasome activation must be tightly regulated, since, if prolonged, it could aggravate the outcome of several inflammation-driven diseases, such as cryopyrin-associated periodic syndrome, arthritis, atherosclerosis, type 2 diabetes, Alzheimer's disease, inflammatory bowel diseases (IBDs), including Crohn’s disease (CD), ulcerative colitis (UC), and cancer [8], whose onset and development is mostly a consequence of multiple risk factors. Among inflammasomes, the NLRP3 pathway is the best studied today, featuring both protective and pathogenic abilities within the gastrointestinal (GI) tract [9]. In this review we aim to summarize the current knowledge of the patho-physiological aspects of NLRP3 inflammasome activation in the GI tract and the potential therapeutic approaches considered nowadays as alternatives to conventional protocols in patients with inflammatory-driven GI disorders, laying particular emphasis on IBD and cancer.

## Emerging mechanisms of NLRP3 inflammasomes in gut homeostasis

The NLRP3 inflammasome is expressed in both innate immune cells and intestinal epithelial cells. To date, three different models of NLRP3 inflammasome activation have been described, namely canonical, non canonical and alternative activation [10]. Canonical activation of NLRP3 involves two consecutive steps: the priming step and the activation step. The priming step, triggered by microbial constituents such as lipopolysaccharides (LPS) or endogenous cytokines, induces the expression of NLRP3 and pro-IL-1β, through the Toll-like receptor 4 (TLR4)-nuclear factor kappa light chain enhancer of activated B cells (NF-κB) pathway. The activation step, which culminates with the cleavage of Caspase-1, occurs as a result of NLRP3 direct or indirect perception of numerous structurally different stimuli and/or changes in intracellular dynamics. Examples include muramyl dipeptide, hemolysins, pore-forming toxins, such as nigericin, bacterial and viral nucleic acids, the fungal wall components zymosan and mannan, adenosine triphosphate (ATP), metabolic stress, alterations in cell volume, changes in calcium ion flux as well as exogenous stimuli or environmental irritants such as cholesterol and uric acid crystals, silica, asbestos, calcium phosphate, alum, serum amyloid A [11]. These indirect exogenous NLRP3 inducers share a common feature, all converging toward a potassium efflux, the key step in the canonical pathway, that culminates in the release of mature cytokines through GSDMD- and NINJ1-mediated membrane pores [12]. Several intermediates have been hypothesized to bridge the potassium efflux to NLRP3 activation, such as lysosomal destabilization, increased levels of reactive oxygen species (ROS), oxidized mtDNA (ox-mtDNA), trans Golgi rupture, microtubule trafficking mediated by acetylated tubulin, dynein and Histone deacetylase 6 (HDAC6) complex machinery, that is essential to convey NLRP3 molecules to the perinuclear microtubule- organizing center (MTOC) [10], where binding to NIMA Related Kinase 7 (NEK7) protein occurs for full activation. Post-translational modifications (PTMs) are required for NLRP3 activation, such as phosphorylation, ubiquitination and sumoylation [13]. Non canonical NLRP3 activation involves direct binding between cytosolic LPS and human Caspase-4/5. Activated Caspases induce a potassium efflux which, in turn, activates NLRP3 inflammasomes, followed by GSDMD activation, cytokines release and pyroptosis [14]. Alternative NLRP3 inflammasomes, found specifically in human and porcine monocytes but not in macrophages, require the engagement of Caspase-8 upon LPS internalization [15]. In 2011, Lissner and colleagues demonstrated that the NLRP3 inflammasome displays protective functions within the GI tract. Indeed, the modulation of the inflammasome activation maintains intestinal epithelium homeostasis through the regulation of commensal microbiota, while confining the growth of harmful bacteria and so maintaining a symbiotic phenotype [16]. Recently, a number of independent studies suggested that proper intracellular NLRP3, Caspase-1, ASC and IL-1β levels are a prerequisite to maintain intestinal epithelial integrity, limit pathogen colonization and prevent systemic dispersion of commensal bacteria and severe colitis [17, 18]. New angles of evidence were reported by He and colleagues, showing that Caspase3/7-mediated GSDMD cleavage generates a short fragment of 13 kDa which, upon nuclear translocation, activates regulatory T-cells (T_reg_), that guarantee appropriate immunity and physiology of the intestinal epithelium [19]. To strengthen this concept, Jung and colleagues identified NINJ1 as one of the key players in preserving colonic homeostasis by hindering the accumulation of M1 macrophages, that is frequently observed in IBD, and shifting the M1/M2 ratio toward an M2 phenotype [20]. These findings reinforce the importance of innate immunity in preserving gut homeostasis, likely by triggering beneficial responses against potential insults in the GI tract, as well as promoting tissue repair mechanisms following injury [21]. However, there are also circumstances in which a chronic activation of NLRP3 speck is deleterious and might, at least partially, subtend the development of GI-associated disorders, such as IBD or cancer.

## Genetic alterations affecting NLRP3 inflammasome performance in IBD

Inflammatory bowel disease (IBD) includes a group of idiopathic chronic inflammatory disorders occurring in the gastrointestinal tract, that historically embraces two distinct conditions: Crohn’s disease (CD) and ulcerative colitis (UC) [22]. Both alterations are characterized by an abnormal immune reaction against the intestinal flora, seen as a threat in genetically susceptible individuals, that causes tissue damage in the colon and a significant risk of evolution to malignant phenotypes [23]. Data from genome-wide association studies (GWAS) have pointed out several hypofunctional NLRP3 polymorphisms (SNPs) associated with the development of IBD, such as rs10733113 found in CD [24] or rs10754558 found in UC [25], suggesting a protective role of the NLRP3 inflammasome against the pathogenesis of IBD. In line with these observations, SNPs on the IL-18 gene, or on the cytokine receptor (IL-18R1) gene, were also associated with an increased susceptibility to IBD, thus the authors ascribed the onset of this disorder to multiple members of the inflammasome pathway [26]. In addition, the missense R779C variant of NLRP3, a point mutation that enhances its activation and pyroptotic cell death, has been associated with the development of very-early onset IBD in children under 6 years old [27]. Similarly, the NLRP3 gain-of-function mutation D305N has been found in adult patients affected by IBD [28].

## Dietary hallmarks behind the NLRP3 inflammasome: the unexpected missing link underlying gastrointestinal tract pathophysiology

**Fig. 2:**
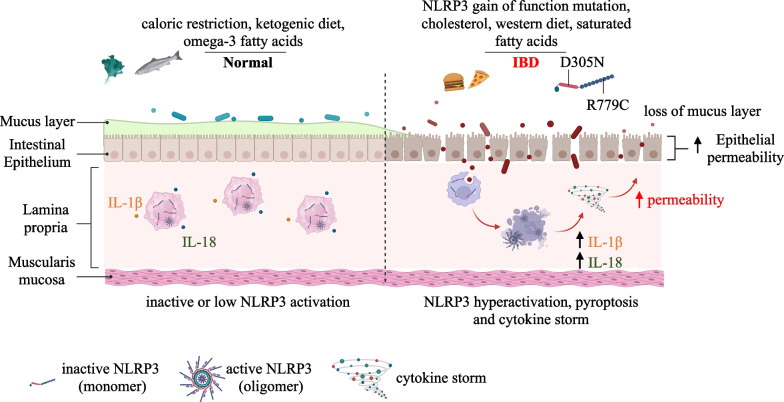
Dietary and genetic factors influencing gastrointestinal homeostasis through the crosstalk between microbiota and NLRP3 inflammasome signaling pathway. Controlled calory intake, ketogenic diet or assumption of omega-3 fatty acids lower NLRP3 inflammasome activation and contribute to the proper homeostasis of the gastrointestinal tract, whereas gain-of-functions, diet rich in cholesterol or saturated fatty acid encourage its activation, recently bridged to inflammation and IBD onset

The molecular mechanism underpinning IBD is largely unknown, but recently, persistent hyperactivation of the NLRP3 inflammasome by colonic macrophages has been considered crucial for the onset and progression of this disease [29]. Some hints may arise from nutritional aspects. Western diet-mediated NLRP3 inflammasome activation, in human monocytes, mediates systemic inflammation and promotes IBD progression [30]. The NLRP3 inflammasome can also affect the status of the gut indirectly, by reshaping the composition of intestinal microbiota, that is essential both for driving intestinal pathogenesis, such as IBD, and also maintaining gut homeostasis [31]. Vice versa, changes in dietary habits, reflected by alterations in the gut microbiota, may influence inflammasome activity, as reported in several studies. Caloric restriction and a ketogenic diet enhance B-hydroxybutyrate production which, in the liver, inhibits NLRP3 activity [32]. Inflammasome inhibition has been correlated with an omega-3 fatty acids rich diet, demonstrating its harmful role in colitis [33], although protective examples, following its activation, has also been demonstrated upon short-chain fatty acids administration via the GPR43 receptor and potassium efflux [34]. Finally, high cholesterol and saturated fatty acids diets exacerbate NLRP3 activation, thereby promoting colitis, cancer, as well as compromising glucose metabolism [35, 36] (Fig. 2). In the following sections, we highlight studies describing the role of the NLRP3 inflammasome-pyroptosis pathway in IBD and cancer development and progression, as well as NLRP3 protective capacities. We also analyze their potential involvement in the development of promising therapeutic strategies.

### NLRP3 inflammasome involvement in Crohn’s disease

Crohn’s disease (CD) is characterized by lesions and transmucosal inflammation typically located in the distal ileum, although in some cases it might extend to other gastrointestinal districts [37]. The etiology of CD has not been fully elucidated, but several studies reported that it is related to environmental, genetic, immunological and nutritional factors [38]; no specific therapeutic approach is yet available. Common treatments nowadays include the use of 5-aminosalicylic acid (e.g.: 5-ASA; sulfasalazine, mesalamine), immunomodulators (e.g.: 6- mercaptopurine, methotrexate, azathioprine), steroids (e.g.: prednisone, budesonide), anti-tumor necrosis factor-α (TNF-α) antibodies or other biological drugs (e.g.: infliximab, adalimumab) [39–44]. Several side effects have been described in patients undergoing those treatments, such as renal toxicity, hemolytic anemia, hepatosplenic T-cell lymphoma insomnia, diarrhea, nausea and vomiting, pancreatitis, bone marrow suppression, lymphoma, fatigue [45]. Interestingly, the platelets NLRP3 inflammasome, often hyperactivated in CD, is a significant source of pro-inflammatory cytokines IL-1β and IL-18 [46], which could further contribute to the development of intestinal micro-thrombosis, hypercoagulability and thromboembolism [47]. The activation of NLRP3, as well as of its downstream effectors GSDMD, IL-1β and IL-18, is proportional to the severity of intestinal lesions manifested in Crohn’s disease [48]. Mechanistically, available IL-1β signals through the mitogen-activated protein kinase (MAPK) cascade to NF-κB, which, in turn, activates genes encoding pro-inflammatory mediators, chemokines and cytokines [49]. However, clinical trials specifically targeting IL-1β, have not shown definitive benefits in patients with CD. On the contrary, some controversial studies reported protective effects of IL-1β in CD. Independent groups reported that mice with a Gsdmd or IL-1β deficiency developed aggressive intestinal inflammation as compared to control littermates [50]. In addition, exogenous IL-1β offers protection against acute colitis in Gsdmd knockout mice [51]. Thus, more in-depth studies are needed to interpret the role of IL-1β in CD. Like IL-1β, IL-18 is involved in intestinal homeostasis and host-microbe interactions, as a member of the IL-1 family [52]. Upon binding to the receptor (IL-18R), IL-18 signals to the myeloid differentiation primary response gene 88 (Myd88) complex [53] to transcriptionally activate genes producing antiviral and antibacterial mediators, such as interferon-γ (IFN-γ) [54]. An elevated IL-18 concentration is found in patients with CD, which promotes the pathogenic T helper 1 (Th1) response, as well as inducing inflammatory mediators, such as TNFα and chemokines [55]. Further studies pointed out a direct role of IL-18 in promoting goblet cell dysfunction, leading to mucosal barrier breakdown, and so potentially exacerbating CD onset [56]. In vivo data showed that IL-18 inhibition, or IL-18R abrogation from intestinal epithelial cells (IECs), protects mice from Dextran Sulfate Sodium (DSS)-induced colitis [57]. However, like IL-1β, specific IL-18 targeting raises concerns. The administration of free recombinant IL-18 reduced colitis in mice, supporting protective functions of IL-18 in colitis [58]. Together, NLRP3 inflammasome activation and its downstream effectors GSDMD, IL-1β and IL-18, shelter against potential infections and help to maintain intestinal homeostasis. However, uncontrolled activation due to chronic exposure to threats might aggravate intestinal inflammation, offering an ideal phenotype for the onset and development of CD. Further investigations are therefore needed to better elucidate the role of the NLRP3 inflammasome in CD, in the switch between protective versus threatening functions, in order to develop new therapeutic approaches.

### NLRP3 inflammasome involvement in ulcerative colitis

Ulcerative colitis (UC) is characterized by a T helper cell type 2 (Th2)-mediated immune response, characterized by mucosal inflammation in the colon and rectum which causes epithelial barrier dysfunction and ulceration [59]. UC lesions usually originate in the rectum and progress toward the proximal colon, causing inflammation in the colonic mucosa and submucosa, as well as promoting an inflammatory phenotype in nearby districts [60]. Patients with UC are more susceptible to colorectal cancer (CRC) compared to the normal population [61]. Macrophages protect the integrity of the epithelial barrier via the secretion of inflammatory cytokines within the gut [62]. A representative facet in UC is the activation of multiple inflammasomes, including NLRP3, NLRC4, NLRP1, and AIM2, leading to Caspase-1 cleavage, cytokines maturation and pyroptosis [63]. The NLRP3 inflammasome role in UC has been deeply analyzed, and is considered a good candidate for targeted therapy. ROS generation, the best known NLRP3 inducer within the intestinal mucosa of patients affected with UC, has been linked, in several independent studies, to inflammasome activation, with IL-1β and IL-18 levels proportional to the disease severity [64]. Moreover, controversial roles have been assigned to GSDMD in UC. Active GSDMD in the intestinal epithelium seems to mediate UC progression [65], whereas other studies showed opposite results, linking GSDMD to UC restriction by controlling cyclic guanosine monophosphate (GMP)-adenosine monophosphate (AMP) synthase (cGAS)-mediated inflammation [66].

## Role of pyroptosis in IBD

The canonical and non canonical NLRP3 inflammasome pathways culminate in a highly inflammatory form of programmed cell death, named pyroptosis, characterized by swollen cells and large bubbles blowing out from the plasma membrane [67]. The pyroptotic process take place upon cleavage of the N-terminus domain of gasdermin proteins (GSDMs). The N-terminus fraction binds to phosphatidylinositol phosphate and phosphatidylserine residues of the plasma membrane and to cardiolipin, present on the inner and outer bacterial leaflets, auto-assembles into membranes and forms pores, causing ion fluxes. Accumulating pores lead, eventually, to cell death [68]. Biological functions ascribed to pyroptosis range from preventing host infections, through cell lysis, to damage to the organism when a severe and uncontrolled inflammatory response occurs [68]. However, GSDMD-mediated pyroptosis might have contrasting outcomes in IBD development, depending on the type of infecting pathogen and host cell. Patients with IBD and mice models of colitis display high levels of GSDMD in the intestinal epithelial cells [65]. Pharmacological blockade, through small molecule inhibitors or Gly-Pro-Ala (GPA) peptide isolated from fish skin gelatin hydrolysate [69], of the Caspase-1-GSDMD pathway attenuated pyroptotic events, improving colitis [70]. Opposing evidence reported that GSDMD plays protective roles in IBD [51], since genetic ablation of GSDMD aggravated colitis by boosting cyclic GMP-AMP synthase (cGAS)-dependent inflammation [66, 71].

## NLRP3 inflammasome targeted therapy in IBD

Several solutions directly targeting the NLRP3 pathway have been considered to ameliorate IBD symptoms [31]. As a multistep activation pathway it offers multiple therapeutic angles. Regulation checkpoints have been proposed at transcriptional [72, 73], translational and posttranslational level, involving the NLRP3 molecule itself. Translational regulation is often mediated by miRNAs, among which, miR-223 has been shown to limit intestinal inflammation by restraining the NLRP3 inflammasome. Following these findings, nanoparticle-mediated overexpression of miR-223 has been developed to attenuate experimental colitis, NLRP3 activation, and IL-1β release [74]. Ubiquitination is required for NLRP3 activation [7]. In vitro approaches have demonstrated that the ubiquitin inhibitor G5 promotes the deubiquitination of NLRP3 and inhibits its activation and IL-1β secretion [75]. Moreover, Kim and colleague showed that ezetimibe ameliorates NLRP3-IL-1β-triggered inflammation through activation of the autophagy pathway [76]. Other indirect interventions might derive from the natural compound curcumin, adequate intake of probiotics, such as *Akkermansia muciniphila*, milk-derived extracellular vesicles, fraxinellone, a lactone compound, alpinetin, a natural flavonoid, and celastrol, a natural triterpene, capable of suppressing the TLR4-NF-κB-NLRP3 axis, and improving UC remission by reducing the infiltration of inflammatory cells [77–82]. Moreover, direct NLRP3 inhibitors have been developed. Treatment with VI-16, a synthetic flavonoid that tethers NLRP3 and abrogates its binding to Thioredoxin interacting protein (TXNIP), results in a complete NLRP3 inflammasome inhibition, ameliorating colitis [83]; Compound 6, a novel tetrahydroquinoline which complex with the NACHT domain of NLRP3, inhibits NLRP3 inflammasome and attenuates colitis in in vivo mouse model [84]. Oral administration of MCC950, the best characterized direct and selective NLRP3 inhibitor, with a half-maximal inhibitory concentration (IC_50_) within the nanomolar range, Oridonin, which competes for binding to NEK7, or INF39, an irreversible inhibitor of the ATPase domain of NLRP3, have shown efficacy against colitis in animal models [85–88]. OLT1177 (Dapansutrile), developed by Olatec in 2012, was the first specific NLRP3 inhibitor to successfully pass phase II (Clinical Trials Identifier: NCT01636141) in patients with colitis [67, 89]. More recently, IFM-2427 (later named DFV890) and NT-0167 compounds, patented by IFM Therapeutics and NodThera, respectively, both in phase I clinical trial, show promising results in the treatment of inflammatory diseases and fibrosis [31]. Lastly, RRx-001, a novel dinitroazetidine small molecule, has display therapeutic function for NLRP3-driven colitis by covalent binding to Cys 409 of NLRP3 via its bromoacetyl group [90]. Efforts are also being made to synthesize compounds that target other inflammasome components, such as the monoclonal antibody against IL-18, GSK1070806, developed by GlaxoSmithKline and currently in phase II (Clinical Trials Identifier: NCT03681067). The alcohol addiction treatment drug, disulfiram, exhibited a potent anti-pyroptotic activity by inhibiting the GSDMD pore-forming function, resulting an excellent candidate for IBD treatment [31] (Fig. 2). Intensive research is focused on targeting the adaptor protein ASC and Caspase-1. Although no tests have been conducted as yet in IBD, ZyVersa Therapeutics have developed a monoclonal antibody, IC 100, against ASC, that is able to attenuate the inflammatory response in multiple sclerosis and acute lung injuries [31]. In addition, promising results are reported for Fc11a-2, a Caspase-1 inhibitor [91].

## The double-edged sword of NLRP3 inflammasome activation in the GI tract: tumorigenesis and suppression

Aberrant inflammation plays a significant role in tumorigenesis of the GI tract. The effector cytokines, IL-1β and IL-18, released during chronic inflammation, induce cancer cell proliferation in a paracrine and autocrine manner [92]. Epithelial gastric cells-derived IL-18 binds to IL-18R, that is overexpressed on the gastric cancer cell membrane, and inhibits Caspase-8-mediated apoptosis while promoting tumor proliferation [93]. Immunosuppression occurs due to the presence of myeloid-derived suppressor cells (MDSCs) and CD4^+^ T cells within the tumor microenvironment (TME) [94, 95]. The presence of NLRP3 inflammasome-dependent IL-1β in the TME of GI cancers is instrumental in suppressing the immune response by recruiting MDSCs which, in turn, secrete inhibitory cytokines and induce T_reg_ cells to exert their immunosuppressive activity [87]. Furthermore, IL-1β drives CD4^+^ T cells to the TME, inducing IL-22 release and thus supporting a pro-tumorigenic niche [96]. IL-18 triggers immunosuppression through different mechanisms. IL-18 has been shown to inhibit natural killer (NK) cells tumoricidal functions by enhancing programmed cell death (PD1) expression and downregulating cluster of differentiation 70 (CD70) [97], two features that incite GI cancer growth. IL-1β offers active contributions to the angiogenesis process. High IL-1β levels have been linked to hyperneovascularization, as they induce the production of pro-angiogenic factors, such as vascular endothelial growth factor-A (VEGF) and hepatocyte growth factor (HGF) [97]. Cancer cell invasion and migration underpin the metastatic cascade. Key events in this process are mediated by IL-1β and IL-18 signaling, as both cytokines accelerate the expression of vascular cell adhesion molecule 1 (VCAM1) in hepatic sinusoidal endothelial cells (HSECs), assisting the adhesion of cancer cells [98]. Cancer cells tend to lose epithelial markers and acquire mesenchymal traits, a process named the epithelial-to-mesenchymal transition (EMT) [99]. It has been shown that IL-1β downregulates E-cadherin while enhancing Zinc finger protein (SNAIL) expression, thus supporting the EMT [99]. However, the NLRP3 inflammasome also orchestrates antitumorigenic tasks in a context- and tissue-dependent fashion.

### Involvement of the NLRP3 inflammasome in esophageal cancer

Esophageal cancer (EC) displays an incidence correlated with geographical areas [100]. High concentrations of nitrosamines in the environment trigger intracellular ROS causing the canonical NLRP3 inflammasome response [101]. EC tissues exhibit elevated levels of active NLRP3 compared to normal tissues [102], that are proportional to the stage and aggressiveness of the disease [98]. Genetic deletion or overexpression of NLRP3 led to the inhibition or induction of cell migration and invasion, respectively [103]. Thus, these data imply pro-tumorigenic functions of the NLRP3 inflammasome within the EC environment.

### Involvement of the NLRP3 inflammasome in gastric cancer

According to the current statistics for cancer-related death worldwide, gastric cancer (GC) is the fourth cause of cancer death in both genders [104]. A significant percentage of GC arises after stomach colonization by Gram-negative *Helicobacter pylori* (*H. pylori*) through the involvement of the TLR2/NLRP3/Caspase-1/IL-18 axis, thus re-shaping gastric immunity [105]. The non-neoplastic genotype of gastric cells relies, at least partially, on the expression of miR-22 and mucin 1 (MUC1), two players that prevent *H. pylori*-induced gastritis and gastric carcinogenesis by inhibiting NLRP3 activation [106, 107]. Of note, carriage of multiple SNPs in the IL-1β gene seems to exert a synergistic increase in the risk of GC when *H. pylori* infection is present [106]. Like *H. pylori*, infection by *Mycoplasma hyorhinis* (*M. hyorhinis*, *M. hy*) fosters IL-1β secretion, which underpins GC cell migration and invasion, in an NLRP3-dependent manner [108]. Wang and colleagues showed that NLRP3 mediates small mother against the decapentaplegic (SMAD) signaling pathway which, in turn, downregulates E-cadherin and promotes the EMT in gastric cancer cells [109]. of human IL-1β promotes gastric tumorigenesis in transgenic mice [110], whereas a reduced tumor volume has been observed in IL-18-silenced nude mice [111]. In view of the higher levels of IL-18 in the gastric tumor microenvironment, two main outcomes arise. Firstly, Caspase-8-mediated apoptosis is dampened, supporting an anti-apoptotic pathway in gastric cancer cells [112]. Secondly, while PD1 expression is enhanced on NK cells, CD70 results downregulated on tumor cells, leading to a decreased cytotoxicity of NK cells [111]. Although the overarching trend of these data suggested a pro-tumorigenic function of the NLRP3 pathway in GC, a more recent genetic profiling revealed that GC is characterized by the absence of GSDMD, whose overexpression significantly inhibits the growth and the proliferation of cancerous cells by arresting the S to G2/M phase transition [113]. Moreover, overexpression of lncRNA, A disintegrin and metalloproteinase with thrombospondin motifs 9 (ADAMTS9)-antisense RNA 2 (AS2) increases the levels of NLRP3, promoting GC cell death [114].

### Involvement of the NLRP3 inflammasome in colorectal cancer

The development and progression of CRC relies on chronic inflammation of the intestinal mucosa but several mechanistic gaps in the knowledge linking inflammation to CRC still exist. However, since chronic inflammation of the intestinal mucosa is the primary vehicle which ultimately leads to CRC, a growing number of studies, albeit partly discordant, have shown involvement of the innate immunity, in particular a misregulated NLRP3 activation, in the onset of this disease [115–118]. While some studies show a tumor suppressive role of NLRP3, others demonstrate a pronounced negative impact. Immunohistochemistry and microarray analysis of the NLRP3 pathway have shown it to be up-regulated in patients affected by CRC, with significantly higher levels of ASC, Caspase-1 and IL-1β than in control tissues [119]. The amplitude of NLRP3 activation seems to be correlated with the expression of *bona fide* EMT markers such as vimentin, N-cadherin, and matrix metallopeptidase 9 (MMP9), reflecting the stage of the disease [120]. CRC is also characterized by elevated levels of 5-hydroxytryptamine (5-HT), known as serotonin [121]. In 2021, Li and colleagues demonstrated that 5-HT enhances NLRP3 activation and IL-1β secretion, via the ion channel receptor binding (5-hydroxytryptamine receptor 3A; HTR3A)-calcium/calmodulin-dependent protein kinase II (Ca^2+^/CaMKII complex) axis, providing further evidence that a 5-HT-NLRP3-positive loop encourages CRC progression [122]. In addition, *Porphyromonas gingivalis*, a pathogen commonly associated with the development of CRC, sustains NLRP3 activation and pro-inflammatory cytokines release from hematopoietic cells [112]. Several studies reported that inflammatory disorders, mediated by an elevated production of IL-1β and IL-18, play a critical role in the pathogenesis of CRC by re-shaping the immunological niche [123, 124]. A further confirmation supporting the pro-tumorigenic role of NLRP3 was obtained in a study led by Wang et al., showing a synergistic cooperation between the mammalian target of rapamycin (mTOR)-Ribosomal protein S6 kinase beta-1 (S6K1)-MAPK and NLRP3 signaling pathways, ultimately promoting the invasion of CRC cells [125]. Finally, several SNPs found in the NLRP3 gene have been linked to poor prognosis in CRC-affected patients [126]. CRC cells express significantly less of the pro-apoptotic protein GSDMD compared to non-neoplastic surrounding cells, allowing them to escape cell death programs [127]. Although the majority of authors in the literature lean towards pro-tumorigenic effects of NLRP3 inflammasome, there are examples of remission symptoms and less body loss in a CRC experimental mice model upon NLRP3 activation [128]. Some groups reported that mice with deficient inflammasome components were more susceptible to CRC than the control group, and showed accelerated tumor growth proportionally to attenuated levels of IL-1β and IL-18, the latter mediating NK cell tumoricidal activity [129]. This suggests that these cytokines might have both detrimental and beneficial effects, depending on the tissue context.

### Involvement of the NLRP3 inflammasome in hepatocellular carcinoma

Hepatocellular carcinoma (HCC) accounts for more than 90% of primary tumors of the liver worldwide [130]. Recently, Wei and colleagues described a possible involvement of the NLRP3 inflammasome in HCC development and progression, after analyzing the mRNA and protein expression level in human HCC tissues and adjacent tissues. Surprisingly, very low expression was detected in HCC tissues, whose level was inversely correlated with advanced stages of the disease [131]. Likewise NLRP3, Caspase-1 resulted downregulated in HCC tissues compared to adjacent non neoplastic cells [132]. The administration of estrogen, such as 17β-estradiol, upregulates NLRP3, which represses HCC cell invasion and migration. The anti-tumorigenic role of the NLRP3 inflammasome pathway in HCC has been reported in several other studies, where extrinsic pathways trigger the activation of NLRP3, thus causing pyroptosis of tumorigenic cells [133]. In sharp contrast, other teams demonstrated that NLRP3 activation coordinates pro-tumoral activities. The tumor-suppressor miR-223 and natural compounds, such as luteoloside and anisonamide, dampen intracellular ROS and suppress NLRP3 activation, modulating HCC cell proliferation and metastasis [134–137]. In 2020, Brocker and colleagues demonstrated that the proliferator-activated receptor (PPAR)-Gm15441 lncRNA axis downregulates NLRP3 activation, and Caspase-1 and IL-β cleavage, controlling inflammation-driven HCC [138]. Furthermore, NLRP3 depletion in HCC model was reported to increase the cytotoxic ability of NK cells on tumor cells [139]. The role of GSDMD and NINJ1, both found overexpressed in HCC, is still under debate [140]. Collectively, these findings unveil contradictory functions for the NLRP3 inflammasome in the pathogenesis of HCC, hence further investigations are essential to fully decipher the connection between HCC onset and NLRP3 activity.

### Involvement of the NLRP3 inflammasome in cholangiocarcinoma

Cancer arising within the bile ducts from bile epithelial cells (BECs), or cholangiocarcinoma (CCA), is typically incurable at diagnosis, but still a rare form of cancer in the Western world, occurring in 0.5–2 people per 100,000 per year [141]. Inflammation of the bile ducts, raw food, bacterial parasites (such as *Helicobacter bilis* and *Helicobacter hepaticus*), viral hepatitis and alcoholic liver disease are risk factors [142]. The development of cholangiocarcinoma has been linked to a lower NLRP3 expression in BECs. An insufficient immunity NLRP3-mediated response to chronic biliary inflammation may underpin the development of carcinogenesis [143]. Although these studies propend for a protective role of NLRP3 against bile carcinogenesis, further efforts are needed to bridge this pathway to the onset of cholangiocarcinoma.

### Involvement of the NLRP3 inflammasome in gallbladder cancer

Gallbladder cancer (GBC), a highly aggressive adenocarcinoma, involves the biliary system, and the survival rate of patients with advanced stage at diagnosis is less than one year [144]. NLRP3 was shown to be upregulated in GBC samples, whose level was positively correlated with the proliferation marker Ki-67. This evidence merges with the presence of active Caspase-1, IL-1β and IL-18 release and pyroptosis, which assist GBC progression [145]. Moreover, the NLRP3 inflammasome drives the phosphorylation of protein kinase B (AKT), extracellular signal-regulated kinases 1/2 (ERK1/2), and cyclic adenosine monophosphate (cAMP) response element-binding (CREB) protein, to further induce cancer growth [146].

### Involvement of the NLRP3 inflammasome in pancreatic ductal adenocarcinoma

The unfavorable prognosis of pancreatic ductal adenocarcinoma (PDA) is associated with high expression and uncontrolled NLRP3 inflammasome activation, high levels of IL-1β, cell proliferation and epithelial mesenchymal transition (EMT)-induced cancer cell invasion, which limit therapeutic options and interfere with patient survival [147, 148]. The expression level of NLRP3 is correlated with the TNM stage; in patients with advanced stage PDA the threshold level established for non-neoplastic adjacent tissues is usually exceeded [149]. Interestingly, this over-activation of NLRP3 matches the downregulation of tumor suppressor long noncoding RNA XLOC_000647, a common hallmark in PDA [150]. An additional NLRP3-mediated feature in the PDA onset is platelet aggregation, which further enhances pancreatic cancer progression and lymph node invasion [151].

## Pyroptosis involvement in GI cancers

Pyroptotic cell death is gaining increasing attention in the field of tumorigenesis nowadays, in view of the opposing evidence of its involvement in the proliferation or inhibition of cancer growth. For tumor associated macrophages (TAMs), whose NLRP3-pyroptosis pathway drives cancer cells to proliferate, accurate therapeutic interventions that specifically target pyroptosis in cancer cells should be considered. Little mechanistic evidence allowing these two different pyroptosis pathways to be discerned has emerged so far. Hepatitis B virus X protein (HBx) is a risk factor for the development of HCC [152]. HBx has been shown, by triggering hepatocytes NLRP3-mediated pyroptosis, to simulate an intrahepatic oxidative stress environment, which underpins HCC onset [153]. Moreover, tumor hepatocytes display upregulated levels of long noncoding RNA SNHG7, which induces the miR34a/SIRT1 pathway, strictly connected to the pyroptosis program via the canonical NLRP3 inflammasome pathway [154]. Low levels of GSDMD in GC are inversely correlated with the expression of cyclin-dependent kinase 2 (Cdk2)/cyclin A2 complexes, fostering the transition from S to the G2 phase, thus accelerating GC cell proliferation [155]. In vivo experiments with GSDMD injection showed a smaller size and lower weight of GC tumors than those injected with the vehicle [147]. CRC shows elevated levels of lncRNA RP1-85F18.6, which obstructs GSDMD activation and pyroptotic cell death [66]. However, although indirectly, overexpression of miR-21-5p stimulates the pyroptosis of CRC cells via the NLRP3-GSDMD axis [155]. Pyroptosis also exhibits an inhibitory influence on PDA. Overexpression of macrophage stimulating 1 (MST1) protein enhances ROS production, which leads to inhibition of the proliferation, migration and invasion of cancerous cells by promoting pyroptosis [156]. Wang and colleagues recently, showed that an alteration in the expression of miR-497 triggers GSDMD-mediated pyroptosis of EC cells [157]. However, more mechanistic studies are required to fully exploit the pyroptotic cell death pathway in the field of tumorigenesis.

## NLRP3 targeted pharmacological interventions in GI cancers

**Fig. 3:**
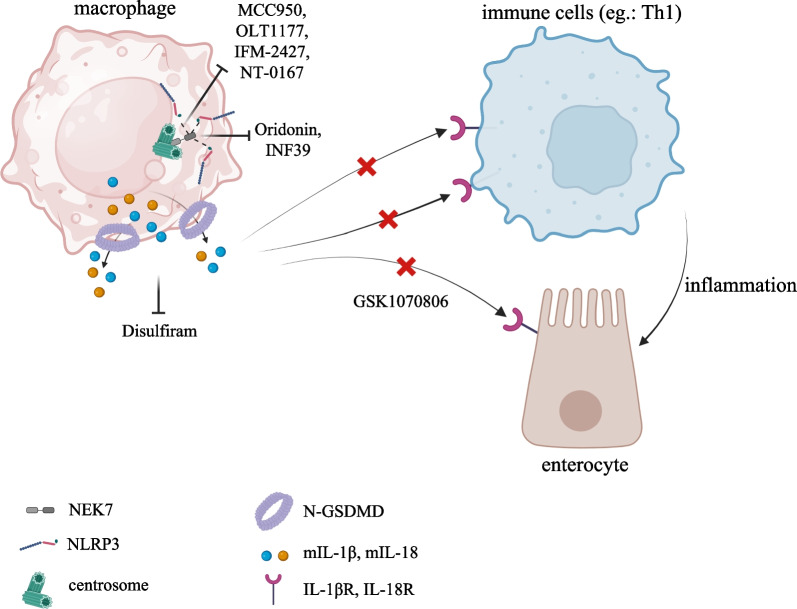
Novel therapeutical approaches targeting NLRP3 inflammasome activation. Several compounds targeting NLRP3-pyroptosis pathway at different stage of activation, able to prevent the activation of Th1 cells and gut inflammation, are currently under clinical trials. MCC950, OLT1177, IFM-2427 and NT-0167 interfere with NLRP3 oligomerization; Oridonin and INF39 hinder the binding between NLRP3 and NEK7; Disulfiram is a potent inhibitor of GSDMD-mediates plasma membrane pores and pyroptotic cell death

**Fig. 4:**
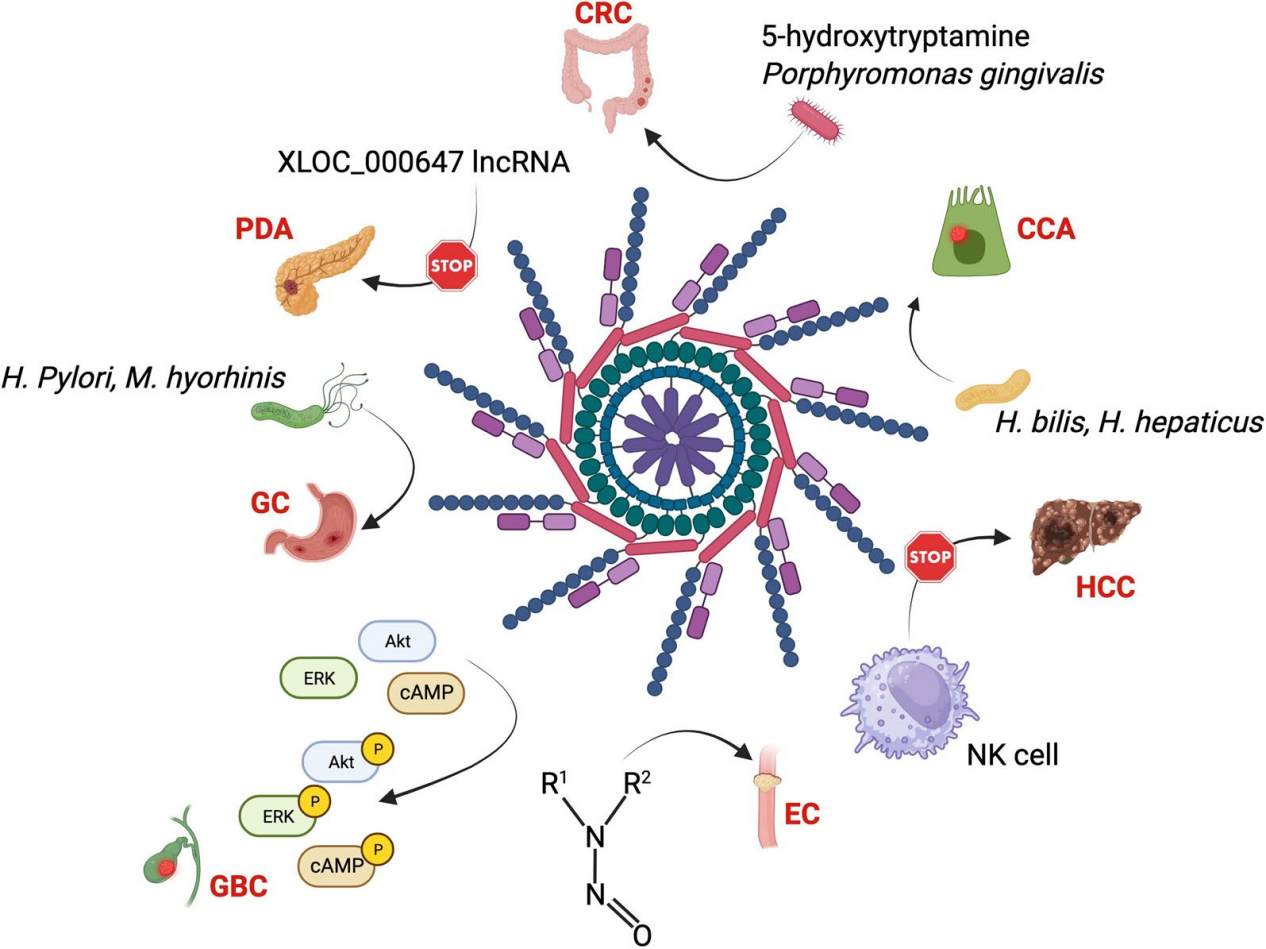
Schematic of NLRP3 inflammasome involvement in GI tract cancers. Accumulating evidences suggest that NLRP3 inflammasome activation underpins the onset of several cancers in the gastrointestinal tract. Environmental nitrosamines-mediated NLRP3 activation has been hypothesized to be involved in the EC. NLRP3 activation functions as immunosuppressor in HCC modulating the tumoricidal activity of NK cell. *Helicobacter bilis* (*H. bilis*) and *Helicobacter hepaticus* (*H. hepaticus*) induce CCA via NLRP3 inflammasome, whereas high 5-HT level and *Porphyromonas gingivalis* colonization promote CRC. Expression of lncRNA XLOC_000647 prevents PDA by dampening NLRP3 activation. *H. pylori* and *M. hyorhinis* trigger GC upon NLRP3 activation. Finally, NLRP3 activation fosters the phosphorylation of AKT, ERK and cAMP signaling pathway which supports GBC onset

The growing evidence coupling a fine-tuning of NLRP3 inflammasome activation to GI cancers (Fig. 3) also emphasizes its therapeutic promise as clinical target. A wide range of agents has now been developed, including small molecule inhibitors, antagonists and monoclonal antibodies for GI cancer therapy, tested in preclinical and clinical trials. NLRP3 inflammasome offers multiple angles of targetable options, which can be classified as specific or not specific NLRP3 small molecule inhibitors. Nowadays, five compounds addressing specifically NLRP3 protein have been employed in cancer treatment, named: MCC950, so far the best characterized inhibitor in several contexts, CY-09, Oridonin, OLT1177 and Tranilast. Although structurally different, they all converge to the NACHT domain, blocking ATP hydrolysis and ASC oligomerization, thus preventing inflammasome activation [119]. Recently, two independent groups reported, for the first time, the efficacy of the administration of MCC950 in PDA. By applying the MCC950 compound on NLRP3 inflammasome-induced cultured pancreatic cancer cell lines, Yaw and colleague showed a reduction of cell viability [158], whereas intraperitoneal injection on mice implanted of Panc02 cancer cells manifested a drastic suppression of platelets Caspase-1 activity, usually upregulated in pancreatic cancer [151]. Additionally, MCC950 has been shown to affect proliferation, to decrease mRNA levels of IL1β and IL18 and to induce apoptosis in HCT116 colorectal cancer cell line as well [159]. PDA has been subjected to a study involving the CY-09 small molecule as well. Yang and colleagues, precisely, reported advantageous outcomes in PDA background upon CY-09 deliver, which strongly dampened the migration and invasion of cancerous cells [160]. There are no other experimental evidences of the use of the CY-09 inhibitor in GI cancers, so far. The inhibitor Oridonin, that targets the Cys 279 residue in the NACHT domain of NLRP3, and its analogs, have been tested in a variety of cancers, including CRC, PDA, GC and EC. Interestingly, Oridonin might represent a potential agent inducing apoptosis of the CRC and human gastric cancer cisplatin-resistant SGC7901/DDP cells through ROS/JNK/c-Jun axis and Caspase activation, respectively [161, 162]. Albeit the mechanistic link with NLRP3 pathway is still missing, by inhibiting cytoskeletal protein LASP1 and PDLIM1, Oridonin induces apoptosis of EC cells, thus arresting cancer growth [163]. Similarly, in pancreatic gemcitabine resistant cancer cell, the LRP1/ERK/JNK signaling is susceptible to Oridonin, which promotes apoptosis and overcomes drug resistance [164]. Further, Oridonin sensitizes HCC cells to the anticancer effects of Sorafenib administration by targeting the AKT pathway [165]. Neither clinical nor pre-clinical applications of OLT1177 compound have emerged in the GI cancer treatment at this time; instead, Tranilast inhibitor influences tumor growth and ameliorates fibrosis in CRC [166] and GC [167]; moreover, it suppresses esophageal cancer stem cells proliferation [168]. Although the above mentioned evidences, summarized in Fig. 4, report intriguingly angles to develop innovative GI cancer trials based on the inflammasome activation, deeper insights into the molecular mechanism of these drugs is highly demanded.

## Involvement of the NLRP3 inflammasome in other type of cancers: perspective to speck-targeted therapeutical approaches

**Fig. 5:**
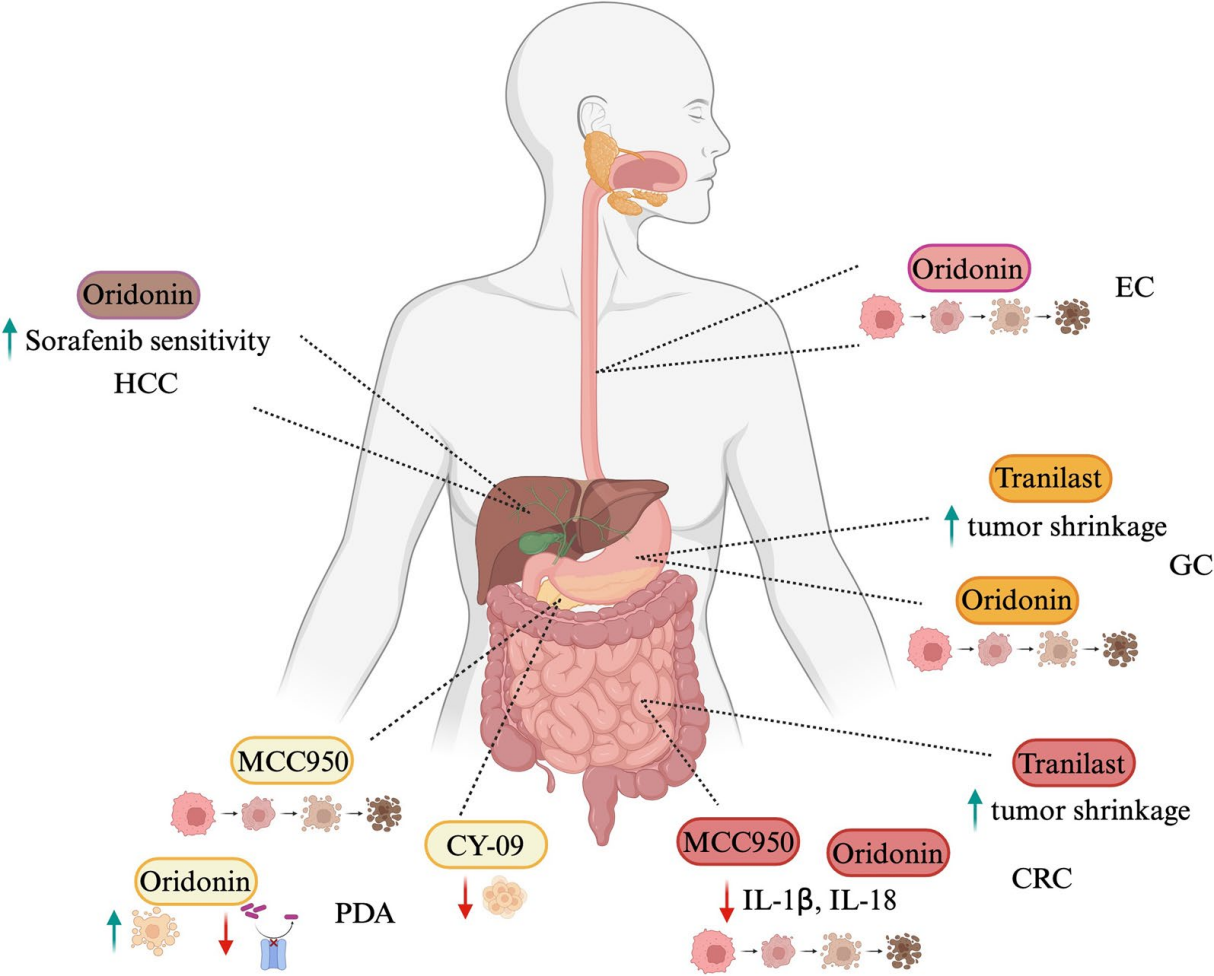
NLRP3-designed therapeutical strategies in GI cancers. Targeted NLRP3 inhibitors employed in the treatment for GI cancers encompass, nowadays, the following compounds: MCC950, Oridonin, CY-09 and Tranilast. MCC950 has been mostly applied in the management of PDA and CRC, where it has shown efficacy in inducing apoptosis of neoplastic cells, concomitantly with IL-1β and IL-18 reduction within the TME. Oridonin is currently under investigation for the treatment of several GI cancers, including HCC, used in combination with Sorafenib, CRC, GC, EC and PDA, found to induce cell death and, specifically in PDA context, to revert drug resistance. The effectiveness of CY-09 inhibitor has been revealed in PDA background, being able to dampen cellular proliferation. Tranilast showed its efficacy in GC and CRC shrinkage

Aberrant NLRP3 inflammasome activation has been recently recognized to play decisive role in the development and progression of several type of cancers, beside those belonging to the GI tract. Although TAMs within breast cancer (BC) microenvironment retain surprisingly low level of intracellular NLRP3, this aggressive neoplasm, which still represents the leading cause of mortality in woman, is characterized by an abundant population of CAFs, which promptly sense DAMPs and trigger the immune response through a robust activation of the NLRP3 inflammasome [169]. Moreover, immunohistochemistry sections of BC tissues showed unc-51-like autophagy activating kinase 1 (ULK1) downregulation, thus further contributing to NLRP3 inflammasome activation through ROS-mediated intervention, which, in turn, assists BC growth [170]. Zheng and colleague found that the integration of CY-09 to the classic gemcitabine treatment dampens tumor resistance by blocking IL-1β/EMT/Wnt/β-catenin signaling pathway in BC [171]. Moreover, NLRP3 knockout mice, orthotopically implanted with metastatic breast cancer cell line (E0771), upon treatment with OLT1177 compound, displayed reduced MDSCs, increased CD8^+^ T cells and NK populations within the microenvironment, increased immunotherapy efficacy, tumor reduction and improved survival [172]. Finally, Oridonin-Loaded Nanoparticles and Tranilast administration have been reported to reduce BC cell migration and invasion, alongside tumor progression, by modulating nuclear factor erythroid 2–related factor 2 (Nrf2) signaling and C-X-C motif chemokine receptor 4 (CXCR4) expression, respectively [173, 174]. The most frequent cancer hitting lungs is the non-small-cell lung cancer (NSCLC), with its 85% of frequency in industrialized countries [175]. NSCLC-derived exosomal tripartite motif containing 69 (TRIM69) protein has been shown to switch TAMs toward a pro-tumoral phenotype by physically interacting and downregulating abhydrolase domain containing 5 (ABHD5) protein, which, in turn, exacerbates NLRP3 inflammasome activation and, consequently, inducing cellular proliferation and invasion [176]. Prolonged NLRP3 speck formation favors lung metastasis spread by altering the ability of NK cells to modulate the tumor [98]. In this context, the inhibitors Tranilast and Oridonin have shown to inhibit cell invasion and to enhance irradiation-induced DNA damage and death of NSCLC cells, respectively [177, 178]. Polymorphisms in the NLRP3 gene have been linked to the development of malignant melanoma [179], with an activation amplitude correlating with the stage of the disease; constitutive inflammasome activation is often found in late stages of melanoma, surrounded by elevated IL-1β level. Currently, OLT1177 and Tranilast inhibitors represent two promising pharmacological candidates in this setting. The combination of OLT1177 and dexamethasone affects the nuclear and mitochondrial isoforms of the signal transducer and activator of transcription 3 (STAT3), capable to shrink tumor growth [180]; alternatively, Tranilast, although though indirect links with NLRP3 inflammasome, has been proposed to inhibit melanoma cell proliferation by activating CD8^+^ T cells [181]. Ovarian cancer (OC) is the seventh most commonly diagnosed cancer among women [182]. NLRP3 expression has been found overexpressed in OC cells, which, usually, correlates with a poor prognosis [183]. In a study led by Luborsky and colleagues, it has been reported a significantly higher expression of Caspase-1, IL-1β and IL-18 in OC tissues compared to normal samples [184]. Therapeutical strategies targeting the NLRP3 inflammasome in OC involve the use of Oridonin, which, through the suppression of the mTOR pathway, arrests OC spread [185], and Tranilast, which enhances the sensitivity of tumor cells to Cisplatin [186]. Hyperactivation of NLRP3-AKT axis, and its IL-1β/NF-kB p65 downstream pathway, has been detected in glioma cells and held responsible for cancer proliferation and migration [187, 188]. The involvement of the NLRP3 inflammasome has been also recently appreciated in multiple myeloma progression, where an abundance of β_2_-microglobulin in the serum drives TAMs-derived NLRP3 speck formation and pro-inflammatory cytokines release. Nanomolar concentration of MCC950 inhibited the β_2_-microglobulin-triggered Caspase-1 activation and IL-1β maturation. Moreover, in vivo experiment showed a reduced tumor size upon MCC950 administration [189]. Oncogenic mutants of kristen rat sarcoma viral oncogene homolog (KRAS) lead to ROS production, NLRP3 activation, IL-1β production and myeloproliferation phenotype in a myeloid leukemia mouse model by varying the proportion between cytoplasmic MYC proto-oncogene/tumor protein p53 (c-myc/TP53) and B-cell lymphoma 2/Bcl-2 Associated X-protein (bcl-2/bax) [190, 191]. The Oridonin inhibitor derivate, named HAO472, is currently under phase I clinical trial for treating acute myelogenous leukemia [192]; In addition, the concomitant administration of Oridonin and valproic acid (VPA) promotes cell death in in vitro experiments involving HL-60 leukemic cells [193]. Lastly, Oridonin effectively reverses the drug resistance of cisplatin and imatinib, thus displaying pronounced anti-leukemia effects [194]. NLRP3-mediated inflammation drives carcinogenesis in head and neck squamous cell carcinoma (HNSCC) [195], where NLRP3 protein, whose level correlates with cancer invasiveness, has been found overexpressed [196]. Delivery of MCC950 in HNSCC mice remarkably reduced NLRP3 inflammasome activation measured as mature IL-1β release; treated mice also displayed lower tissue resident MDSCs, T_reg_ cells and TAMs as well as increased CD4^+^ and CD8^+^ T cells, which may denotes a novel approach for HNSCC therapy [197]. Endometrial cancer unveils increasing incidence in developed countries [198]. Mechanistically, it has been shown that estrogens upregulate NLRP3 expression via estrogen receptor β (ERβ), which foster cancer cells proliferation; accordingly, NLRP3 knockdown inhibited cancer growth [199]. Conversely, independent in vivo study claimed opposite conclusions, assigning, through MCC950 employment, protective roles to NLRP3 inflammasome in the management of endometrial cancer [200]. Recently, Santos‑Ruiz laboratory revealed synergic antitumor effects of Oridonin and doxorubicin in human osteosarcoma cells, although mechanistic evidences that link this outcome with NLRP3 inflammasome are still missing [197]. Similarly, a reduction of tumor proliferation has been observed treating nasopharyngeal carcinoma (NPC) cells with Tranilast inhibitor, which conveys to the NF-kB pathway [201]. New insights have been provided into the mechanisms of NLRP3 inflammasome as a potential target for the treatment of prostate cancer (PCa). By counteracting the upregulation of the NLRP3 inflammasome in PCa cells with the Caspase-1 inhibitor Z-YVAD-FMK, authors have been able to impair PCa growth through in vitro and in vivo experimental approaches [202] (Fig. 5).

## Conclusion

Studies on inflammasome and pyroptosis mechanisms have made substantial progress in the last decade. The present review aims to summarize the latest understanding of the role of the NLRP3 inflammasome and pyroptosis in gastrointestinal inflammation and cancer. The NLRP3 inflammasome is not only involved in a wide variety of common diseases, but displays contextualized functions, in a tissue-specific manner, with contradictory phenotypic outcomes. Several insults have been proposed to activate NLRP3, although the molecular mechanism remains unclear. Discovered by the Jürg Tschopp research team in 2002, the NLRP3 inflammasome mediates the first line of defense against pathogens through an intracellular multistep process which involves several participants responsible for the activation of inflammatory responses. The complex clusters the NLRP3 protein, which senses the danger, adaptor protein ASC, and pro-Caspase-1, later converted into the bioactive form, which triggers the formation of mature cytokines together with the activation of the pyroptosis executor GSDMD [7]. The biologically active IL-1β and IL-18 mediate a series of responses that can ultimately shape the local environment, with divergent repercussions, including those involved in the onset, progression, as well as curtailment of IBD and GI cancers. IBD and GI tract cancers have become a global health problem with a complex physiology, involving several research macro-areas features spanning from genetics to immunity, microbiology and tissue morphology and physiology. A controlled NLRP3-pyroptosis axis from colonic macrophages might restrict pathological threats. However, IBD onset is often correlated with unrestricted activation of inflammasomes, piquing the curiosity of several biotech companies which are enthusiastically performing high-throughput screening to find novel NLRP3 and pyroptosis inhibitors and improve the quality of life of affected people. Both the NLRP3 inflammasome and GSDMD-assisted pyroptosis are involved in all the steps of tumorigenesis: proliferation and/or survival, immunosuppression, angiogenesis and metastasis [203]. Moreover, the response to therapy is affected as well, based on the NLRP3 activation level [204]. Although cancer studies on NLRP3 and pyroptosis are still in an early stage, the rapid development of research technology, nanomaterials, natural and chemotherapeutic drugs, holds great potential in this field. The approach aims to target the two main components of the tumor: the microenvironment, mainly characterized by TAMs and cancer-associated fibroblasts (CAFs), and the cancer cells. TAMs are a subset of tumor-infiltrating macrophages that sustain the abnormal growth of neoplastic cells, which, together with CAFs, are capable of inducing the activation of the canonical NLRP3 inflammasome and secreting pro-inflammatory cytokines, feeding the tumor [205]. On the other hand, cancer cells might be susceptible to induced pyroptotic cell lysis. The development of novel strategies able to: 1. obstruct cytokines pathways; 2. alter post-translational modifications of the inflammasome to dampen its activation and 3. synergistically switch the proliferation phenotype to a pro-apoptotic one, should be considered nowadays as a therapeutic advantage in specifically killing cancer cells. However, challenges to be considered for a pyroptosis-based approach to cancer therapy might arise due to the genotypic and phenotypic heterogeneity of various tumors. For instance, mutations or altered expression levels of inflammasome components could affect the efficacy of the treatment. In addition, cancer cell metabolites could influence the pyroptotic outcomes. Finally, the NLRP6-pyroptosis axis has been recently linked to the liquid‒liquid phase separation (LLPS) research field, through which intracellular membraneless compartments accelerate biological reactions, such as antiviral immune responses, by sequestering specific proteins and avoiding unrelated ones [206]. However, the mechanism underpinning LLPS-mediated inflammasome activation is unclear and whether the NLRP3 inflammasome requires LLPS to function is not known. Thus, deeper molecular insights into the mechanism of NLRP3 inflammasomes and pyroptosis are needed to definitively link the inflammasome with pathophysiology of the GI tract and to achieve a clinical translation to be potentially employed in conjunction with a personalized medicine approach.

## Data Availability

Not applicable.

## Abbreviations

5-HT: 5-Hydroxytryptamine
5-FU: Fluorouracil
ABHD5: Abhydrolase domain containing 5
ADAMTS9: A disintegrin and metalloproteinase with thrombospondin motifs 9
AIM2: Absent in melanoma-2
AKT: Protein kinase B
AS2: Antisense RNA 2
ASC: Apoptosis-associated speck-like protein containing a CARD
ATP: Adenosine triphosphate
BC: Breast cancer
Bcl 2: B-cell lymphoma 2
Bax: Bcl-2 Associated X-protein
BECs: Bile epithelial cells
c-myc: Cytoplasmic MYC proto-oncogene
CAFs: Cancer associated fibroblasts
CaMKII: Calmodulin-dependent protein kinase II
CCA: Cholangiocarcinoma
CD: Crohn disease
CD70: Cluster of differentiation 70
cAMP: Cyclic adenosine monophosphate
Cdk2: Cyclin-dependent kinase 2
cGAS: Cyclic guanosine monophosphate-adenosine monophosphate synthase
CRC: Colorectal cancer
CREB: CAMP response element-binding
Cys: Cysteine
CXCR4: C-X-C motif chemokine receptor 4
DAMPS: Damage-associated molecular patterns
DSS: Dextran sulfate sodium
EC: Esophageal cancer
EMT: Epithelial-to-mesenchymal transition
ERβ: Estrogen receptor β
ERK1/2: Extracellular signal-regulated kinases 1/2
GBC: Gallbladder cancer
GC: Gastric cancer
GI: Gastrointestinal
GPA: Gly-Pro-Ala peptide
GSDMD: Gasdermin D
GSTO1: Glutathione S-transferase omega 1
GWAS: Genome-wide association study
*H. bilis*: *Helicobacter bilis*
*H. hepaticus*: *Helicobacter hepaticus*
*H. pylori*: *Helicobacter pylori*
HBx: Hepatitis B virus X
HCC: Hepatocellular carcinoma
HDAC6: Histone deacetylase 6
HNSCC: Head and neck squamous cell carcinoma
HGF: Hepatocyte growth factor
HTR3A: 5-Hydroxytryptamine receptor 3A
IBD: Inflammatory Bowel Disease
IC_50_: Inhibitory concentration
IECs: Intestinal epithelial cells
IL-1β: Interleukin-1β
IL-18: Interleukin-18
IL-18R: Interleukin-18
IL-1RA: Interleukin-1 receptor antagonist
IFN-γ: Interferon-γ
KRAS: Kristen rat sarcoma viral oncogene homolog
LASP1: LIM and SH3 domain protein 1
LPS: Lipopolysaccharides
LLPS: Liquid–liquid phase separation
lncRNA: Long non-coding RNA
MAPK: Mitogen-activated protein kinase
MDSCs: Myeloid-derived suppressor cells
miR: MicroRNA
MMP9: Matrix metallopeptidase 9
MTOC: Microtubule-organizing center
mTOR: Mammalian target of rapamycin
MTX–TMPs: Methotrexate-loaded tumor-cell-derived microparticles
MST1: Macrophage stimulating 1
MUC1: Mucin 1
*M. hyorhinis*: *Mycoplasma hyorhinis*
MYD88: Myeloid differentiation primary response gene 88
NACHT: NAIP (neuronal apoptosis inhibitor protein), C2TA (MHC class 2 transcription activator), HET-E (incompatibility locus protein from Podospora anserina) and TP1 (telomerase-associated protein)
NEK7: NIMA-related kinase 7
NF-κB: Nuclear factor kappa light chain enhancer of activated B cells
NINJ1: Ninjurin 1
NK: Natural killer cells
Nrf2: Nuclear factor erythroid 2–related factor 2
NLR: NOD-like receptor
NLRC: Nucleotide-binding oligomerization domain, Leucine rich Repeat and CARD domain containing
NLRC4: Nucleotide-binding oligomerization domain, Leucine rich Repeat and CARD domain containing 4
NLRP: Nucleotide-binding oligomerization domain, Leucine rich Repeat and Pyrin domain containing
NLRP1: Nucleotide-binding oligomerization domain, Leucine rich Repeat and Pyrin domain containing 1
NLRP3: Nucleotide-binding oligomerization domain, Leucine rich Repeat and Pyrin domain containing 3
NLRP6: Nucleotide-binding oligomerization domain, Leucine rich Repeat and Pyrin domain containing 6
NPC: Nasopharyngeal carcinoma
NSCLC: Non-small-cell lung cancer
OC: Ovarian cancer
PAMPS: Pathogen-associated molecular patterns
PCa: Prostate cancer
PD1: Programmed cell death
PDA: Pancreatic ductal adenocarcinoma
PDLIM1: PDZ and LIM domain protein 1
PPAR: Proliferator-activated receptor
PRR: Pattern recognition receptors
PTMs: Post-translational modifications
ROS: Reactive oxygen species
S6K1: Ribosomal protein S6 kinase beta-1
SMAD: Small mother against decapentaplegic
SNAIL: Zinc finger protein
STAT3: Signal transducer and activator of transcription 3
TAM: Tumor-associated macrophages
Th1: T helper 1
Th2: T helper 2
TLR4: Toll-like receptor 4
TME: Tumor microenvironment
TNF-α: Tumor necrosis factor-α
TP53: Tumor protein p53
T_reg_: Regulatory T cells
TRIM69: Tripartite motif containing 69
TXNIP: Thioredoxin interacting protein
UC: Ulcerative colitis
ULK1: Unc-51-like autophagy activating kinase 1
VEGF-A: Vascular endothelial growth factor-A
VCAM1: Vascular cell adhesion molecule 1
VI-16: Flavonoid VI-16
VPA: Valproic acid
